# South West Vascular Surgeons

**Published:** 1991-09

**Authors:** 


### South West Vascular Surgeons

### Abstracts of Meeting held at Gloucester in February 1991

### INFLAMMATORY ANEURYSMS OF THE ABDOMINAL AORTA: THE BRISTOL EXPERIENCE

**W. G. Tennant, S. G. W. Tennant, M. G. Wyatt, R. N. Baird, M. Horrocks**
Bristol Royal Infirmary

Inflammatory aneurysms of the aorta can be difficult to detect and hazardous to repair. We present Bristol's experience over five years.

Inflammatory aneurysms occurred in younger patients and were associated with significantly more pain. Plasma viscosity was raised in inflammatory disease, and was a good indicator. Radiological investigations were of doubtful value.

Involvement of other organs in fibrosis led to major modification of operative technique in several cases.

Repair of inflammatory aneurysms took longer, and patients were given more blood and blood substitutes peroperatively. Bifurcated grafts were more often required.

Following repair of inflammatory aneurysms patients had a higher morbidity and mortality, although survivors had the same long term outlook as patients with simple aneurysms.

This study illustrates the unique problems that are posed in the surgical care of inflammatory aneurysms.

### HAS VASCULAR SURGERY IMPROVED THE OUTLOOK FOR PATIENTS WITH ABDOMINAL AORTIC ANEURYSMS?

**C. A. C. Clyne**
Torbay Hospital

A retrospective survey of all known abdominal aortic aneurysms presenting to Torbay Health District, between 1982 and 1990, has been carried out to determine the impact of elective repair on emergency presentation and mortality. Data was obtained from the operating theatre register, the vascular register, the OPCS (Petersfield, Hants) and from mortality and morbidity meetings.

The overall number of known aneurysms in the District in 1982 was 24, compared to 60 in 1989, but the percentage presenting with rupture however fell in those years from 83% to 29%. The total recorded deaths from aortic aneurysm has not significantly risen since the mid 1980's (1982/14 — 1986/28 — 1989/27) and this mortality in 1989 was made up of a fairly constant figure of 30 day deaths following elective aneurysm repair, deaths following aneurysm rupture and those diagnosed at post mortem.

These figures encourage us to operate on most aneurysms presenting electively and suggest that such an aggressive policy may reduce community aneurysm rupture rates, and strengthens the argument for screening for aortic aneurysms.

### DUODENAL OBSTRUCTION AFTER ABDOMINAL AORTIC ANEURYSM REPAIR

**P. M. Lamont, J. Collin**
John Radcliffe Hospital, Oxford

Duodenal obstruction is a rare post-operative complication of abdominal aortic aneurysm repair. Out of only four previously reported cases, two were post-mortem findings and the other two were managed surgically by gastrojejunostomy and duodenojejunostomy respectively. We present here three further cases of duodenal obstruction after aneurysm repair, one due to intramural haematoma and two due to the superior mesenteric artery syndrome. All three cases were managed conservatively with parenteral nutrition and an expectant policy, with a successful outcome in each case after 7-12 days of restrained surgical temptation.

### CONTAINED RUPTURE OF AN ABDOMINAL AORTA ANEURYSM: CASE REPORT AND REVIEW OF THE LITERATURE

**M. Koppikar, M. G. Wyatt, A. R. Baker**
Frenchay Hospital, Bristol

A 75 year old woman developed a sudden onset of severe abdominal pain. An abdominal ultrasound was performed two weeks after the onset of the pain and demonstrated a 7cm abdomial aortic aneurysm (AAA) and a seperate left upper quadrant mass. A CT scan performed at six weeks confirmed the diagnosis of a high infrarenal AAA and showed the left upper quadrant mass to be a large contained rupture which was eroding the vertebral body at the level of the upper pole of the left kidney. A surgical opinion was sought and urgent repair arranged. Via a thoraco-abdominal approach the aorta was clamped above the renal arteries to enable access to the sizable contained rupture. Her post-operative recovery was uneventful. Although AAA's are common, rupture is usually fatal if surgery is delayed. The combination of a 6 week old contained rupture with successful surgical repair is unusual. The literature is reviewed and the management discussed.

### TWO CASES OF ANASTOMOTIC AORTIC ANEURYSMS

**S. M. Plusa**
Queen Alexandra Hospital, Portsmouth

We have recently treated 2 patients with anastomotic aortic aneurysms.

A 67 year old man complained of claudication 6 years after an aortobifemoral graft. CT Scan confirmed an anastomotic aortic aneurysm which was treated with an interposition graft.

A 74 year old man presented with back pain due to retroperitoneal bleeding from an aneurysm at the distal aortic anastomosis 11 years after emergency aneurysm repair.

Anastomotic aneurysms may present with pain or ischaemic symptoms. The factors most often implicated are infection and aspects of surgical technique.

### POPLITEAL EXPLORATION FOR ACUTE ISCHAEMIA

**C. Ranaboldo, S. Parvin and S. Darke**
Poole General Hospital

Exploration of the popliteal artery is a technically demanding procedure which is rarely performed for patients with acute limb ischaemia. Within an 18 month period 15 popliteal vessels were explored in 13 patients.

Only one patient died, having previously undergone repair of a ruptured abdominal aortic aneurysm. 12 of 15 limbs (80%) were salvaged. 2 of the 3 amputations were performed in patients with a preceding history of occlusive arterial disease. Immediate reconstruction was attempted in only two cases, without success. No other complications arose.

Popliteal embolectomy from this small series appears to produce an excellent rate of limb salvage in a selected group patients.

### POPLITEAL ENTRAPMENT — A SNAKE IN THE GRASS?

**D. C. Wilkins**
Plymouth General Hospital

Three cases of popliteal artery entrapment are presented. Two presented few problems in diagnosis, but one had a few clinical or radiological diagnostic features.

The first case was a 20 year old Polytechnic student who presented with acute ischaemia of one lower leg. Arteriography showed the typical displacement associated with popliteal entrapment. Successful revascularisation of the symptomatic side was carried out and, later, release of the contra-laterel asymptomatic side.

The second case was a 45 year old man who presented with an acute onset of claudication. Arteriography showed a blocked lower superficial femoral and popliteal artery, but with little displacement on arteriography. His age, lack of risk factors and distribution of disease were the main factors in making the diagnosis. The contra-lateral side was explored and a typical entrapment released.

The third case was a 20 year old man who presented with acute claudication and demonstrated typical radiological changes. Successful release and revascularisation of the affected side was carried out. The other side was normal.

The second case illustrates that diagnosis of this condition is not always easy and a high index of suspicion should be maintained.

### ANGIOSCOPY IN ARTERIAL THROMBOEMBOLECTOMY

**Philip D. Colman**
John Radclife Hospital, Oxford

Improved technology in fibreoptics and the development of fine flexible catheters has prompted a renewed interest in vascular endoscopy. Potential applications are still being identified. The influence of angioscopy in arterial thromboembolectomy will be discussed based on experiences at Harbor-U.C.L.A. Medical Centre, California. The technique of semi-closed embolectomy, intially described by Fogerty suffers from several limitations. In our experience, angioscopy overcomes many of those limitations. It allows the surgeon to inspect the vessel and its contained thrombus and embolus, monitor the embolectomy procedure and confirm complete clearance of the vessel.

### PROSTHETIC ABOVE KNEE FEMORO POPLITEAL BYPASS — SHOULD THE VEIN BE SAVED FOR ANOTHER DAY?

**G. Sutton, D. R. Allen, J. Thompson, A. D. B. Chant, J. H. Webster**
Royal South Hants Hospital, Southampton

We have reviewed all above knee prosthetic grafts inserted over a 4 year period to January 1989. 68 grafts were inserted, 8 for rest pain and 60 for claudication. Grafts were followed up 6 monthly with duplex scanning. Median follow up was 18 months (2-60 months). 6 patients died with patent grafts. 9 grafts occluded; of these, 4 had a successful below knee bypass using vein, 2 had no suitable vein, therefore a Biograft was used and 3 had no procedure.

If a superficial femoral occlusion is not amenable to angioplasty, bypass to the above knee popliteal segment with a prosthetic graft is safe, quick and preserves the vein for a distal procedure if required.

### IMPORTANCE OF GRAFT REVISION TO ACHIEVE PATENCY AFTER EARLY OCCLUSION OF FEMOROPOPLITEAL VEIN BYPASS

**D. R. Jones, J. J. Earnshaw, T. R. Magee, A. H. Davies, R. N. Baird, M. Horrocks**
Bristol Royal Infirmary

Multiple re-operations following early occlusion of femoropopliteal vein bypass occured in 43 cases. Analysis of the first re-operation revealed 21 graft revisions and 22 graft thrombectomies alone. Secondary patency was achieved in 18 vein bypasses (41.9%), 8 vein grafts remained occluded, the outcome in 17 grafts (40%) was major amputation.

**Table 1:**
**Limb salvage after reoperation for early occlusion of femoropopliteal bypass**

| | amputation | limb salvage |
|---|---|---|
| revision | 5 | 16 |
| thrombectomy | 12 | 10 |

Chi square = 4.25 p 0.05

If thrombectomy alone is performed at the first re-operative procedure for early femoropopliteal vein graft occlusion there is a significantly lower limb salvage rate than if the graft is revised.

### WHAT IS THE PROGNOSIS FOR 'NON VASCULAR LEG PAIN'?

**K. Varty, J. Van Dorpe, J. A. St.Johnston, W. B. Campbell**
Royal Devon & Exeter Hospital

During a 3 year period 525 patients were referred to our Vascular Clinic with lower limb pain. We have studied the progress of 55 (10.5%) in whom arterial insufficiency was not the cause. These patients were invited for follow up 1-4 years later and 50 attended (91%). There were 33 men and 22 women aged 22-86 years (median 67).

Fourteen patients (29%) had seen other specialists resulting in the following diagnosis:— spinal stenosis (3), sciatica (4), lumbar spondylosis (2), peripheral neuropathy (3), multiple sclerosis (2), osteo-arthritis of the hip (1) and Mortons neuroma (1).

Thirty five patients (70%) remained undiagnosed. Eleven had improved, 16 were unchanged and 8 had deteriorated. The mean pain score was low (3.5/10) with a high mean quality of life score (32/40).

One patient had significant arterial disease not noted at the original assessment. Eight others had mild arterial disease, noted originally, but which did not account for their symptoms.

In summary a minority of patients referred with non vascular leg pain have troublesome symptoms due to orthopaedic or neurological conditions. The majority have relatively mild symptoms, remain stable or improve and no definite diagnosis is made. Our clinical assessment rarely missed significant arterial disease.

### ENDOSCOPIC THORACIC SYMPATHECTOMY

**D. C. R. Adams, S. J. Wood, B. Heather, R. N. Baird, K. R. Poskitt**
Cheltenham General Hospital, Gloucester Royal Hospital and Bristol Royal Infirmary

Thoracic sympathectomy is the treatment of choice for upper limb hyperhidrosis and may be of some benefit in Raynaud's phenomenon and peripheral vascular disease. As open surgery using the supraclavicular approach has poor access and the transaxillary route is painful and often requires chest drainage, we have assessed the endoscopic approach.

Thirty upper limbs (18 patients) were treated. Twelve patients suffered with hyperhidrosis, 4 with Raynaud's phenomenon and two with peripheral vascular disease. All but one patient showed a symptomatic improvement. Two patients suffered with temporary Horner's syndrome and five patients required chest drainage. Four patients required endoscopic division of adhesions prior to sympathectomy. Endoscopic thoracic sympathectomy is recommended as a safe and effective method to replace open surgery.

### FITNESS FOR VASCULAR SURGERY AND ANAESTHESIA-CARDIAC RISK STRATIFICATION USING DOPPLER-DERIVED CARDIAC OUTPUT

**J. N. Davies, C. J. Ranaboldo, J. H. H. Webster, A. D. B. Chant**
Royal South Hants Hospital, Southampton

A prospective study of 100 patients undergoing elective vascular surgery was undertaken. Cardiac output at rest and after arm ergometry was measured noninvasively with the ACCUCOM 2 suprasternal Doppler (Datascope Medical Co. Ltd.).

All measurements were blinded from surgeon and anaesthetist, and outcome in terms of cardiac morbidity and mortality monitored postoperatively, and compared with other indices of risk such as Goldman Score.

Doppler-derived Cardiac Output was a good predictor of outcome. The results are discussed.

### CHRONIC MESENTERIC ISCHAEMIA REVASCULARISED BY VEIN GRAFTS

**V. B. Conboy, A. A. Shandall, G. Carolan, I. F. Lane**
University Hospital of Wales, Cardiff

Chronic mesenteric ischaemia is a rare phenomenon with the classic triad of post-prandial pain, weight loss and diarrhoea being masked by other symptoms, leading to extensive investigations and a late diagnosis. Four patients, mean age 55 years at presentation, have undergone revascularisation. Diagnosis was considerably delayed before arteriography revealed occlusion of at least two mesenteric arteries. All underwent aortomesenteric vein bypass with revascularisation of the superior mesenteric artery, plus coeliac axis in two patients. There were no operative deaths and graft patency was confirmed by arteriography or duplex scan. All were symptom free at a mean of 10 months post-operatively.

### REINFORCED VASCULAR GRAFTS: A COMPARATIVE STUDY

**T. R. Magee, P. G. Niblett, W. B. Campbell**
Royal Devon & Exeter Hospital

Reinforced vascular grafts are designed to resist compression or flexion forces, for example in axillo-bifemoral or below knee femoro-popliteal reconstruction.

This study compared eight types of 6mm reinforced grafts (5 PTFE and 3 Dacron). On a specially designed rig, graft diameter and flow were measured during compression by a standard series weights up to 3.6 kg, and on another rig, grafts were flexed through 135°.

Meadox Microvel graft resisted compression best (p  0.001), but there were no important differences between the other grafts (Vascutek, Bard EXS, Goretex Standard, Thin Wall, and Removable Ring, Impra Flex Standard and Thin Wall.

Flexion produced no significant change in flow through the grafts.

In addition, five general surgeons were asked to score the ease of removal of reinforcement, passage of sutures, and the tendency for sutures to cut out of each graft. Removal of reinforcement and passage of sutures were easier with the PTFE grafts, but Dacron was more resistant to sutures cutting out.

This study shows that the grafts resist flexion and compression forces well. Their different handling characteristics and data on patency at different sites should remain the main determinants in choice of grafts.

### ANGIOPLASTY IN THE DIABETIC. IS IT THE SAME?

**A. H. Davies, S. E. Cole, T. R. Magee, D. Jones, R. N. Baird, M. Horrocks**
Bristol Royal Infirmary

Angioplasty is an important tool in the armamentarium of the clinician dealing with atherosclerotic disease. Diabetic patients with occlusive disease pose special problems.

Between 1980-87, 285 non diabetic and 48 diabetic lower limbs have undergone single site angioplasty. No difference in site was found, but a significant difference in indication for treatment (x2=33 p  0.001). At a minimum time of follow up of three years the overall success rate in diabetic patients n=48 was 25% and 43% in non diabetics n=285 (p  0.05 x2=5.06). However if outcome is compared with indication there was no significant difference.

Initial results, with angioplasty are reasonable but the longterm outcome is poor. This study suggests that diabetes has no specific influence on the outcome of angioplasty.

